# Bioethanol production using vegetable peels medium and the effective role of cellulolytic bacterial *(Bacillus subtilis)* pre-treatment

**DOI:** 10.12688/f1000research.13952.2

**Published:** 2018-05-03

**Authors:** Salman Khan Promon, Wasif Kamal, Shafkat Shamim Rahman, M. Mahboob Hossain, Naiyyum Choudhury

**Affiliations:** 1 Department of Mathematics and Natural Sciences, BRAC University, Dhaka, 1212, Bangladesh; 2 United Surgical (BD) Ltd, Kadda, Gazipur, 1702, Bangladesh; 3 Bangladesh Atomic Energy Regulatory Authority (BAERA), Dhaka, 1207, Bangladesh

**Keywords:** Bioethanol, yeast, cellulolytic bacteria

## Abstract

**Background:**  The requirement of an alternative clean energy source is increasing with the elevating energy demand of modern age. Bioethanol is considered as an excellent candidate to satiate this demand.

**Methods:** Yeast isolates were used for the production of bioethanol using cellulosic vegetable wastes as substrate. Efficient bioconversion of lignocellulosic biomass into ethanol was achieved by the action of cellulolytic bacteria ( *Bacillus subtilis*).  After proper isolation, identification and characterization of stress tolerances (thermo-, ethanol-, pH-, osmo- & sugar tolerance), optimization of physiochemical parameters for ethanol production by the yeast isolates was assessed. Very inexpensive and easily available raw materials (vegetable peels) were used as fermentation media. Fermentation was optimized with respect to temperature, reducing sugar concentration and pH.

**Results:** It was observed that temperatures of 30°C and pH 6.0 were optimum for fermentation with a maximum yield of ethanol. The results indicated an overall increase in yields upon the pretreatment of *Bacillus subtilis*; maximum ethanol percentages for isolate SC1 obtained after 48-hour incubation under pretreated substrate was 14.17% in contrast to untreated media which yielded 6.21% after the same period. Isolate with the highest ethanol production capability was identified as members of the ethanol-producing *Saccharomyces* species after stress tolerance studies and biochemical characterization using Analytical Profile Index (API) ® 20C AUX and nitrate broth test. Introduction of *Bacillus subtilis* increased the alcohol production rate from the fermentation of cellulosic materials.

**Conclusions:** The study suggested that the kitchen waste can serve as a raw material in ethanol fermentation.

## Abbreviations

SC1 - yeast isolates from sugarcane juice; DJ1 - yeast isolates from date juice; pH - Negative logarithm of hydrogen ion concentration; °C - Degree Celsius; % - Percentage; CH _3_CH _2_OH - ethanol or ethyl alcohol; YEPD - Yeast Extract Peptone Dextrose; nm - Nanometer; gm - Gram; ml - Milliliter; rpm - Round per minute; v/v - volume per volume; w/v - weight per volume; spp. - Species; *et al*. - And others.

## Introduction

With the aims of protecting the environment and reducing dependence on petroleum and nonrenewable energy sources, the development of renewable energy sources has become increasingly important. Ethanol can be produced chemically from petroleum, and from biomass or sugar substrates fermentation ^1^. Fermentation-derived ethanol (CH _3_CH _2_OH) or ethyl alcohol is commonly known as bioethanol. This organic chemical is a flammable, clear and colorless liquid which can be used as fuel. Other functions of ethanol include its use as a solvent, antifreeze and germicide ^2^.

Several processes of bioethanol production currently exist, such as microbiological production from fermentable organic substrates or carbohydrates by yeast. Fermentation of cellulosic biomass, molasses, vegetable peels or food wastes can be considered as an economical process of bioethanol production ^3^. Bioethanol produced from cellulosic materials by direct conversion is utilized in countries such as Brazil, Canada and, USA ^4^. The economical production of bioethanol requires an easily available supply of inexpensive raw materials. Organic food waste is one of the topmost suitable materials for that process. Solid food wastes from household, restaurants or food processing industries can be obtained as a substrate to be used as fermentation medium for bioethanol production. Food wastes can also be recycled as animal feed and fertilizer after specific treatment.

The foremost focus of this ethanol production technology is the optimized utilization of biomass resources and microbial action on fermentation. One promising technique is the fermentation of lignocellulosic biomass where hydrolysis by specific microbial cellulase enzymes is involved ^5, 6^. Ethanol can be derived from the fermentation of sugar-containing materials. Different yeast varieties are reported for the fermentation of lignocellulosic substrates to produce ethanol ^7, 8^.

The objective of the project is to establish a highly efficient microbial fermentation process by natural yeast isolates to produce bioethanol. Other objectives included the characterization of the microbial strain. It is to be mentioned that ethanol production rate from insoluble lignocellulosic biomass is currently not economical. Therefore, commonly available cellulosic kitchen wastes were used as raw material. Proper treatment of the substrate was done to optimize the fermentation condition which has resulted in a highly efficient and economical production rate. Potential wild-type yeast strains were isolated from date juice, sugarcane juice, grapes, and pineapples. Wild-type yeasts were identified by the biochemical and physiological characterization and taken under comparative studies and experiments to obtain a strain with high productivity. Cellulose degrading bacteria ( *Bacillus subtilis*) was used for pre-treatment of the fermentation media. Cellulolytic microorganism debased celluloses present in the lignocellulosic fermentation media and the degraded materials were easier and more readily available to be fermented by yeast.

## Methods

### Sample collection and isolation

Wild-type yeasts were isolated from sugarcane juice and date juice. Aforementioned sources were collected from the local market and kept for 1 week at room temperature for yeast growth. The samples were inoculated into YEPD (Yeast Extract Peptone Dextrose) broth which is composed of 1% yeast extract (Y1625), 2% peptone (P7750), 2% glucose or dextrose (G8270) (Sigma-Aldrich, St Louis, MO, USA) and the desired volume of distilled water. Cultures from the broth were plated on YEPD agar media and incubated at 37°C for 48 hours. The grown colonies were cultured again on YEPD agar medium under the same growth condition to obtain isolated colonies. After the incubation, the isolated colonies (slant) were preserved at 4°C refrigeration. The culture was maintained by periodic sub-culturing.

### Identification

**Figure 1.**
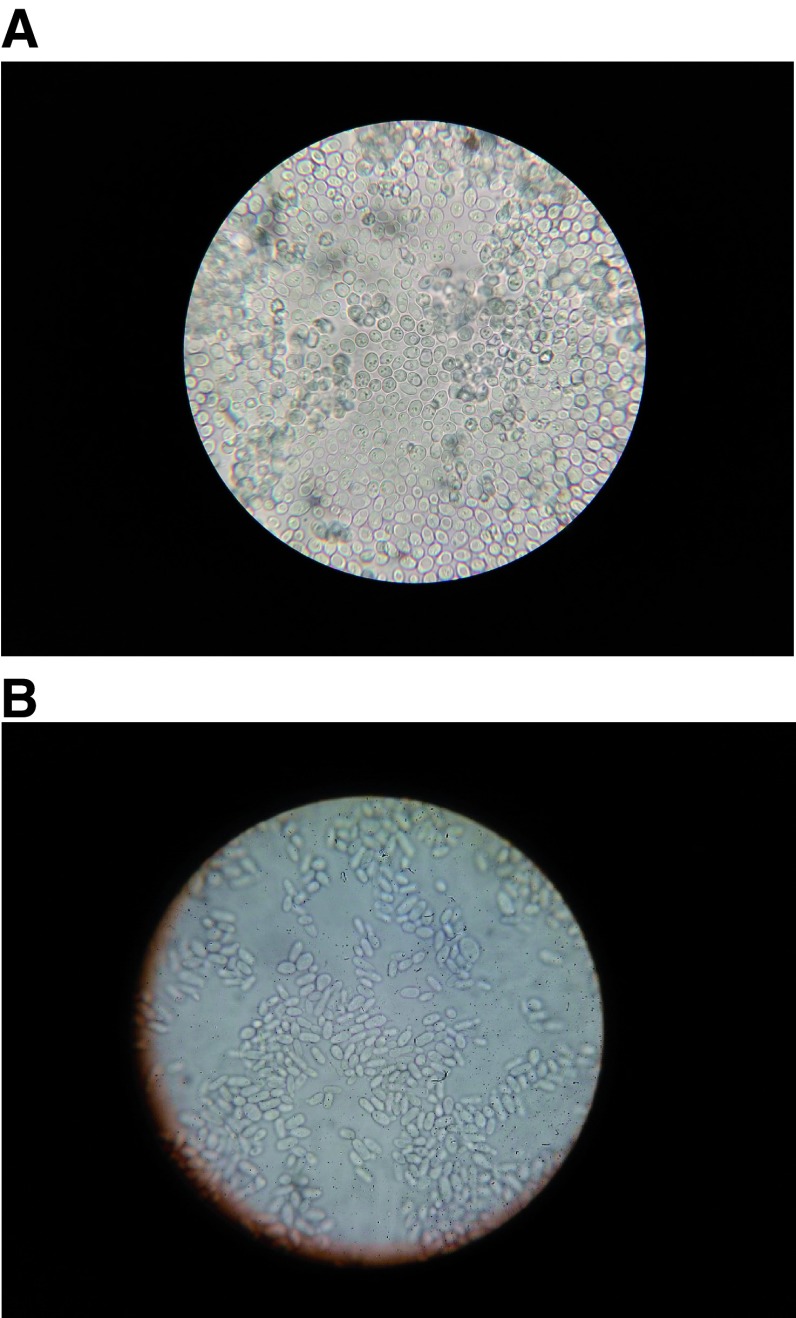
The cell morphology (unicellular ovoid shape, multipolar budding; white and creamy texture) of yeasts under the compound microscope (100X) from sugarcane juice ( **A**) and date juice ( **B**).

A compound microscope (Model-CX-21, Olympus, Japan) was used to observe the cell morphology and the presence of yeasts were confirmed which were isolated from sugarcane juice ( Figure 1A) and date juice ( Figure 1B) (named SC1 and DJ1 respectively). Identification of each isolate of yeast up to species level was carried by the methods demonstrated by Kreger-Van Rij (1984) ^9^ based on the morphology, sporulation and fermentation characteristics, as well as the assimilation of nitrogen and a range of carbon sources. Yeast specific API® identification kit (bioMérieux, Marcy-l'Étoile, France) was also used by inoculating 48-hrs culture broths into the chambers according to the protocol.

### Stress tolerance tests

Ethanol tolerance of yeast isolates was tested by inoculating isolates in YEPD broth supplemented with varying concentrations (5%, 10%, 15% and 20%) of absolute ethanol and incubated at 30°C for 48 hours ^10^. To observe the thermotolerance, the isolates were inoculated in YEPD broth and incubated at different temperatures (25°C, 30°C, 37°C and 44°C) for 48 hours. The growth of the yeast isolates at different pH was observed by inoculating isolates in YEPD broth with different pH (2–10; adjusted by adding drops of basic NaOH or acidic diluted HCl in the solution while reading a pH meter (E-201-C Shanghai Ruosuaa Technology company, China)) and incubated at 30°C for 48 hours. Initial optical densities of each tube during inoculation and optical densities after incubation were measured using the spectrophotometer (UVmini-1240 spectrophotometer, Shimadzu, Kyoto, Japan) at 600 nm against the medium as the blank.

### Fermentation media preparation

Lignocellulosic biomass was used as fermentation medium. Residual waste parts of potato, papaya, pumpkin, the cucumber was used as fermentation medium. These vegetable peels were collected from households (Mohakhali area) and chopped into smaller pieces. Solid wastes (250 gm) were pulverized with 1000 ml water in an electrical blender machine. The blended material was transferred into a beaker and boiled for 10–15 minutes. Hydrochloric acid was added (2 ml) to decrease the pH to avoid bacterial contamination and convert calcium to calcium sulfate salts.

### Preparation of microorganism cell suspensions

Yeast subcultures derived from 24–48 hours old streak plates were inoculated into 10 ml of 0.9% normal saline using a sterile loop. A cell suspension of *Bacillus subtilis* was prepared by inoculating 24 hours’ old culture into 10 ml NaCl (0.85%) saline. The suspensions were made homogenous using vortex machine after inoculation.

### Fermentation of cellulosic media

150 ml fermentation media was added to 500 ml Erlenmeyer conical flasks. Cellulosic media were aseptically inoculated with *Bacillus subtilis* suspension and incubated for 24 hours at 37°C in shaking condition (80 rpm). After the incubation, yeast cell suspension was inoculated and the flasks were cotton plugged and incubated in a rotary incubator (WIS-20R, Wonju-si, Daihan Scientific, Korea) at 30°C for 48 hours in shaking condition (120 rpm). Yeast isolates were inoculated into another set of similar cellulosic media which were not treated with the cellulolytic organism and incubated under the aforementioned fermentation condition.

### Estimation of ethanol

Initial assay of ethanol production rates was performed by volumetric analysis in Conway units ^11^. A fractional distillation set was used to separate ethanol from fermented broths. Samples yielding feasible results were distilled and the ethanol percentages of the distillates were determined by specific gravity using an alcohol meter (5453 Vinometer, LD Carlson, Kent, OH, United States).

## Results

**Figure 2.**
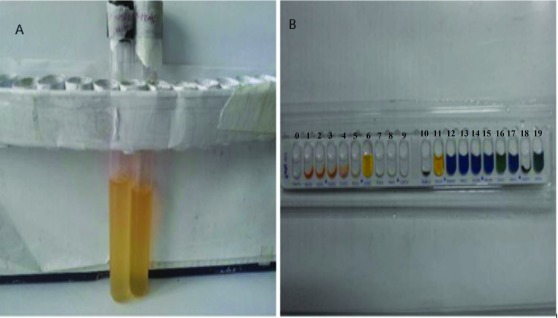
Biochemical tests to identify yeast species. ( **A**) Negative nitrate reduction test indicated by the yellow color (positive test would result in red color change) ( **B**) API 20 C X kit results for different carbohydrates fermentation using API kit after 48 hours. Color in the chamber indicates a positive result, negative results retain the yellow color of the broth. Active ingredients contained in each chamber from left to right: 0 – none, 1 – D-glucose, 2 – Glycerol, 3 – Calcium 2-keto-D-gluconate, 4 – L-arabinose, 5 – D-xylose, 6 – Adonitol, 7 – Xylitol, 8 – D-galactose, 9 – Inositol, 10 – D-sorbitol, 11 – Methyl α-D-glucopyranoside, 12 – N-acetylglucosamine, 13 – D-cellobiose, 14 – D-lactose, 15 – D-maltose, 16 – D-saccharose/Sucrose, 17 – D-trehalose, 18 – D-melezitose, 19 – D-raffinose.

Morphology was visually observed as white and creamy texture, ovoid shape, multipolar budding pattern, under microscope ( Figure 1A and B). Sporulation was confirmed due to the presence of ascospore. Nitrate reduction was not exhibited in the nitrate assimilation test ( Figure 2A). Carbohydrate assimilation tests were conducted using API® 20C test strips (bioMérieux, Marcy-l'Étoile, France) ( Figure 2B). In all cases, positive results were obtained for glucose, galactose, maltose, starch, and fructose ( Table 1). Therefore, carbohydrates and nitrate assimilation test results signified the strong probability of isolates being the species *Saccharomyces cerevisiae*. Yeast isolates from sugarcane juice (SJ1) had a good growth at 25°C, 30°C, and 37°C, but showed poor growth at 40°C and 44°C. Yeast isolates from date juice (DJ1) had a good growth at 30°C, and 37°C, moderate growth at 40°C, but propagated poorly at 25°C and 44°C. Yeast isolate SC1 and DJ1 showed a variable growth result at pH 2–10. Overall, pH 5 and 6 was optimum growth conditions where the isolate SC1 exhibited the highest growth at pH 6 and DJ1 had its best growth at pH 5. All isolates showed excellent growth at 5% and 10% ethanol concentrations throughout the entire 48-hour incubation period ( Table 2).

**Table 1.** Fermentation result of different carbohydrates for sugarcane juice (SC1) and date juice (DJ1) isolate. (Legends: + + Positive, + - Variable, -- Negative).

Carbohydrate	SC1	DJ1
Glucose	+ + (gas)	+ + (gas)
Maltose	+ + (gas)	+ + (gas)
Galactose	+ + (gas)	+ + (gas)
Starch	+ +	+ +
Sucrose	+ -	- -
Fructose	+ + (gas)	+ + (gas)
Trehalose	- -	- -
Lactose	- -	- -
Xylose	- -	- -

**Table 2.** Alcohol production in different physical/chemical condition after 48 hours of fermentation. (Legends: + + Positive, + - Variable, -- Negative).

Isolate	Temperature	48 hours growth			
		Initial O.D.	Final O.D.	O.D. change	Growth
SC1	25°C	0.244	1.184	0.94	+ -
DJ1		0.529	1.401	0.872	+ -
SC1	30°C	0.112	1.540	1.428	+ +
DJ1		0.441	1.103	1.544	+ +
SC1	37°C	0.311	1.42	1.109	+ +
DJ1		0.525	1.436	0.911	+ -
SC1	40°C	0.368	0.524	0.156	+ -
DJ1		0.463	0.754	0.291	+ -
SC1	44°C	0.513	0.493	-0.02	- -
DJ1		0.685	0.532	-0.153	- -
Isolate	Ethanol percentage	48 hours growth			
		Initial O.D.	Final O.D.	O.D. change	Growth
SC1	5%	0.103	1.476	1.371	+ +
DJ1		0.172	1.555	1.385	+ +
SC1	10%	0.261	0.488	0.227	+ +
DJ1		0.338	0.592	0.254	+ +
SC1	15%	0.201	0.314	0.113	+ +
DJ1		0.097	0.151	0.054	+ -
SC1	20%	0.075	0.109	0.034	- -
DJ1		0.191	0.254	0.063	- -
SC1	25%	0.218	0.167	-0.051	- -
DJ1		0.257	0.11	-0.147	- -
Isolate	pH	48 hours growth			
		Initial O.D.	Final O.D.	O.D. change	Growth
SC1	2	0.186	0.066	-0.12	- -
DJ1		0.201	0.009	-0.192	- -
SC1	3	0.303	1.13	0.827	- -
DJ1		0.335	1.276	0.941	+ -
SC1	4	0.390	1.249	0.859	- -
DJ1		0.409	1.44	1.031	+ -
SC1	5	0.179	1.412	1.233	+ +
DJ1		0.164	1.472	1.308	+ +
SC1	6	0.108	1.536	1.428	+ +
DJ1		0.377	1.846	1.469	+ +
SC1	7	0.145	1.337	1.192	+ -
DJ1		0.452	1.441	0.989	- -
SC1	8	0.356	1.53	1.174	+ -
DJ1		0.424	1.572	1.148	+ -
SC1	9	0.243	1.391	0.737	- -
DJ1		0.384	1.264	0.880	- -
SC1	10	0.433	0.398	-0.035	- -
DJ1		0.528	0.522	-0.006	- -

**Figure 3.**
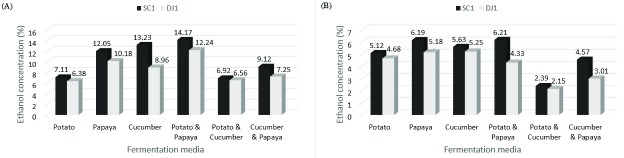
Alcohol production from vegetable peels by yeast isolates SC1 and DJ1 at pH 6. Media treated with *Bacillus subtilis* ( **A**) and No pre-treatment ( **B**).

With fermentation conditions of 30°C incubation temperature with a pH of 6, the highest rate of alcohol production from a cellulosic medium (a mixture of papaya and potato peels pretreated with *Bacillus subtilis*) was 14.17% v/v or 141.7 gm/L (w/v) by yeast isolate SC1 ( Figure 3A; Dataset 1). On the other hand, under the same fermentation conditions, the highest rate of alcohol production using the same cellulosic medium not treated with cellulolytic bacteria was 6.21% v/v or 62.1 gm/L (w/v) by the isolate SC1 ( Figure 3B; Dataset 2). The highest rate of alcohol production from the pretreated potato and papaya media was 12.24% v/v or 122.4 gm/L (w/v) by isolate DJ1 ( Figure 3A; Dataset 1) under a 48-hour fermentation condition at 30°C incubation temperature. The lowest alcohol production rate recorded under the same conditions using the untreated potato and cucumber media (2.15% v/v) by the isolate DJ1 ( Figure 3B; Dataset 2). Fermented media with the highest percentage of alcohol (14.17% v/v) was distilled by a fractional distillation set. This highest percentage was achieved by the yeast isolate SC1 in fermentations condition of 30°C, pH 6 at 120 rpm. The cellulosic media pretreated with *Bacillus subtilis* was distilled after fermentation and the distilled product (one-time distillation) had an ethanol percentage of 52% v/v. In contrast, cellulosic media which was not treated with *Bacillus subtilis* had an ethanol percentage of 12% v/v after the first distillation. Fermentation in other media was recorded highest at 6.23% or 62.3 gm/L (w/v) alcohol production at pH 6 by SC1 isolate after 24-hrs fermentation ( Table 3).

**Table 3.** Alcohol production from defined sugars by yeast isolate SC1 and DJ1.

Isolate	Defined sugar medium	pH	Percentage of ethanol after 24 hours			Avg. gm/L (w/v)	Percentage of ethanol after 48 hours			Avg. gm/L (w/v)
			First round	Second round	Avg.		First round	Second round	Avg.	
SC1	Glucose	6	2.28	2.21	2.24	22.4	2.37	2.31	2.34	23.4
		5	3.35	3.33	3.34	33.4	2.99	2.93	2.96	29.6
DJ1		6	1.91	1.87	1.89	18.9	1.98	1.9	1.94	19.4
		5	3.91	3.67	3.79	37.9	3.87	3.81	3.84	38.4
SC1	Sucrose	6	2.22	2.12	2.17	21.7	2.23	2.15	2.19	21.9
		5	2.71	2.67	2.69	26.9	2.81	2.73	2.77	27.7
DJ1		6	1.74	1.68	1.71	17.1	1.79	1.81	1.80	18
		5	2.21	2.13	2.17	21.7	2.25	2.23	2.24	22.4
SC1	Molasses	6	6.56	5.9	6.23	62.3	5.99	5.79	5.89	58.9
		5	5.37	4.87	5.12	51.2	5.5	5.34	5.42	54.2
DJ1		6	3.3	3.28	3.24	32.4	3.92	3.86	3.89	38.9
		5	4.41	4.27	4.34	43.4	4.95	4.91	4.93	49.3

Alcohol production from vegetable peels by yeast isolates SC1 and DJ1 at pH 6. Media pre-treated with Bacillus subtilis Click here for additional data file. Copyright: © 2018 Promon SK et al. 2018 Data associated with the article are available under the terms of the Creative Commons Zero "No rights reserved" data waiver (CC0 1.0 Public domain dedication).

Alcohol production from vegetable peels by yeast isolates SC1 and DJ1 at pH 6. Media without Bacillus subtilis pre-treatment Click here for additional data file. Copyright: © 2018 Promon SK et al. 2018 Data associated with the article are available under the terms of the Creative Commons Zero "No rights reserved" data waiver (CC0 1.0 Public domain dedication).

## Discussion

Despite the availability of several industrial strains of yeasts, local isolates are usually more adapted to their own climatic condition. In this study, yeasts were isolated from local resources to serve the economic purpose. Utilization of isolated yeasts is an important strategy for the production of bioethanol ^12^. On the basis of the white and creamy appearance of selected isolates on solid media with butyrous colony texture, polar budding and oval cellular shape it can be assumed that isolates are members of *Saccharomyces* spp. from the method described by Boekhout and Kurtzman (1996) ^13^. Fermentation of different sugars by the selected yeast isolates was observed. Yeast isolates from sugarcane (SC1) utilized glucose, maltose, fructose, galactose, starch, sucrose, and arabinose but failed to grow on sorbitol, melibiose, mannitol, trehalose, inositol, xylose and lactose. Yeast isolates from date juice (DJ1) utilized glucose, maltose, fructose, galactose and starch, but failed to grow in trehalose, xylose, sucrose and lactose. The conclusion was further reinforced by biochemical tests performed using bioMérieux’ API® 20C kits. Kit results for SC1 and DJ1 indicated that all of the isolates are *Saccharomyces cerevisiae*. As previous studies by Ramani *et al*. (1998) ^14^ indicate that API® 20C kits have a statistical accuracy of 97% for common yeasts, the conclusions were assumed to be correct. Furthermore, nitrate assimilation tests for all isolates yielded negative results which confirming our hypothesis. Thermotolerance tests also indicated that all isolates (SC1 and DJ1) grew best at 30°C within a 48-hour incubation period; this is also the optimum growth temperature of *Saccharomyces cerevisiae* described by Alexopoulos (1962) ^15^. As for ethanol tolerance, the general trend observed was a decrease in terms of tolerance of all isolates above 10% ethanol concentration signified by a slowdown in growth rate with a near growth stunt at 20%. Teramoto *et al*. (2005) ^16^ demonstrated that members of *Saccharomyces* spp. can tolerate ethanol concentrations of up to 16.5%. However, since the isolates are wild-type *Saccharomyces* yeasts, an average maximum tolerance of 10% ethanol does not mean that they cannot be members of *Saccharomyces* spp. Different growth factors affect the pH tolerance of yeast. It was reported by Ivorra *et al*. (1999) ^17^ that the optimum pH range for ideal growth varies from 4–6 depending on the strain. The cellular structure of yeast has a diverse mechanism to endure pH. In this experiment, yeast isolates SC1 and DJ1 had a variable growth result from pH 2–10. Both of the isolates had excellent growth from pH 4 to 6. However, those isolates were able to grow at all the pH condition, but pH lower than 3 and higher than 7 was not suitable for a good growth. Overall, pH 5 and 6 were optimum growth conditions where isolate SC1 had its best growth at pH 6 and isolate DJ1 had its best growth at pH 5. The ethanol production rate was recorded from the fermentation of different cellulosic media after 24 and 48-hours fermentation. The production rate ranged from 2.15% or 21.5 gm/L to 14.17% v/v or 141.7 gm/L (w/v). Isolate SC1 had the highest rate of ethanol production (14.17% v/v), and isolate DJ1 had the lowest rate of ethanol production (2.15% v/v) in shaking condition at 30°C with a media pH of 6. Ethanol production rate was also observed in shaking condition at 30°C with a media pH of 5. In this condition, isolate SC1 had the highest rate of ethanol production (9.42% v/v) and isolate DJ1 had the lowest rate of ethanol production (2.17% v/v), which surpassed the previous reports ^18– 20^. Ethanol production using kitchen waste media has exceeded the earlier works ^21^. In a study, Nofemele *et al*. (2012) ^22^ demonstrated 7.8% percent ethanol production from sugarcane molasses using *Saccharomyces cerevisiae*. Maximum 9.48% ethanol yield resulted in a similar 2012 study ^23^.

In Bangladesh, five yeast isolates were reported ^24^ to be used for the similar experiments where those isolates (TY, BY, GY-1, RY and SY) had alcohol production rate of 12.0%, 5.90%, 5.80%, 6.70% and 5.80%, respectively at 30°C after 48 hours of incubation. Arapoglou et al. (2010), Wantanee and Sureelak (2004) and Yamada et al. (2009) reported 7–20 gm/L ethanol yield from potato peels ^25^, which is exceeded (46.6–82.7 gm/L) in the present study. Significant elevation of ethanol production rate was observed in the co-fermentation process where cellulosic media were inoculated with cellulolytic bacteria previously. Overall, the method proves the efficiency of the co-fermentation ^26– 28^. The economic advantage of using vegetable peels media over molasses is the recycling process of abundant garbage. On the other hand, molasses has a purchasing value. Addition of nutrition supplements in future endeavors is also recommended ^18^.

## Conclusions

The present study allowed the isolation and characterization of two *Saccharomyces cerevisiae* isolates (SC1 and DJ1) with potential for ethanol production. Yeast isolated from sugarcane juice (named SC1 for this study) showed the highest percentage of alcohol production from cellulosic substrates. Vegetable peels pretreated with cellulolytic bacteria are detected as a suitable fermentation substrate. If the fermentation conditions are optimized, this procedure may be used for large-scale bioethanol production from cellulosic wastes. Scaling up of the experiment can be beneficial for power generation, bioethanol can be used as an alternative to fossil fuels. The raw materials required for the production of bioethanol are cheap and available. It will decrease environmental pollution, pave the pathway towards a proper waste management system and also fertilizers can be produced from the wasted substrate. This study was limited to vegetable peels media without diversification. In future, municipal organic waste may be considered in this regard. Recombinant strains may be employed to optimize the alcohol production rate, too.

## Data availability

The data referenced by this article are under copyright with the following copyright statement: Copyright: © 2018 Promon SK et al.

Data associated with the article are available under the terms of the Creative Commons Zero "No rights reserved" data waiver (CC0 1.0 Public domain dedication). 

**Dataset 1:** Alcohol production from vegetable peels by yeast isolates SC1 and DJ1 at pH 6. Media pre-treated with *Bacillus subtilis*. 10.5256/f1000research.13952.d195639 ^29^

**Dataset 2:** Alcohol production from vegetable peels by yeast isolates SC1 and DJ1 at pH 6. Media without *Bacillus subtilis* pre-treatment. 10.5256/f1000research.13952.d195640 ^30^

## Author information

When the research was carried out, SSR was a MSc student at Biotechnology program, Department of Mathematics and Natural Sciences, BRAC University, and NC was the Coordinator of Biotechnology and Microbiology programmes at Department of Mathematics and Natural Sciences, BRAC University.

## References

[1] Hossain N Zaini JH Mahlia TM: A Review of Bioethanol Production from Plant-based Waste Biomass by Yeast Fermentation. 2017;8(1):5–18. 10.14716/ijtech.v8i1.3948

[2] Licht FO: World Ethanol Market: The Outlook to 2015.Agra Europe Special Report, Tunbridge Wells, UK,2006; Accessed 12 Sep 2017. Reference Source

[3] Peris D Moriarty RV Alexander WG: Hybridization and adaptive evolution of diverse *Saccharomyces* species for cellulosic biofuel production. 2017;10(1):78. 10.1186/s13068-017-0763-7 28360936 PMC5369230

[4] Thenmozhi R Victoria J: Optimization and improvement of ethanol production by the incorporation of organic wastes. 2013;4(5):119–23. Reference Source

[5] Yamada R Nakashima K Asai-Nakashima N: Direct Ethanol Production from Ionic Liquid-Pretreated Lignocellulosic Biomass by Cellulase-Displaying Yeasts. 2017;182(1):229–37. 10.1007/s12010-016-2322-2 27844339

[6] Lee YG Jin YS Cha YL: Bioethanol production from cellulosic hydrolysates by engineered industrial *Saccharomyces cerevisiae*. 2017;228:355–61. 10.1016/j.biortech.2016.12.042 28088640

[7] Chandra RP Bura R Mabee WE: Substrate pretreatment: the key to effective enzymatic hydrolysis of lignocellulosics? 2007;108:67–93. 10.1007/10_2007_064 17530205

[8] Wang J Hu M Zhang H: Converting Chemical Oxygen Demand (COD) of Cellulosic Ethanol Fermentation Wastewater into Microbial Lipid by Oleaginous Yeast *Trichosporon cutaneum*. 2017;182(3):1121–30. 10.1007/s12010-016-2386-z 28130766

[9] Kreger-Van Rij NJ: The Yeast a Taxonomic Study. New York: Elsevier Science Publishing Company.1984;1082 Reference Source

[10] Fakruddin M Islam MA Quayum MA: Characterization of Stress Tolerant High Potential Ethanol Producing Yeast from Agro-Industrial Waste. 2013;1(2):24–34. 10.11648/j.ajbio.20130102.11

[11] Conway RK McClelland J Shapouri H: Comments Concerning the Environmental Protection Agency’s Regulation of Fuels and Fuel Additives: Renewable Oxygenate Requirement for Reformulated Gasoline Proposed Rule. Public Document A-93-49. U.S. Department of Agriculture, Office of Energy;1994; Accessed 15 Sep 2017.

[12] Jayakody LN Ferdouse J Hayashi N: Identification and detoxification of glycolaldehyde, an unattended bioethanol fermentation inhibitor. 2017;37(2):177–89. 10.3109/07388551.2015.1128877 26953525

[13] Boekhout T Kurtzman CP: Principles and methods used in yeast classification, and an overview of currently accepted yeast genera.In: Wolf K, editor. *Nonconventional Yeasts in Biotechnology: A Handbook.*Springer-Verlag, Berlin, Heidelberg;1996;1–99. 10.1007/978-3-642-79856-6_1

[14] Ramani R Gromadzki S Pincus DH: Efficacy of API 20C and ID 32C systems for identification of common and rare clinical yeast isolates. 1998;36(11):3396–98. 9774605 10.1128/jcm.36.11.3396-3398.1998 PMC105341

[15] Alexopoulos CJ: Sub-class hemiascomycetidae, the yeast and leaf-curl fungi.In: *Introductory Mycology.*Second Edition. Toppan Printing Company, Japan;1966;241–58.

[16] Teramoto Y Sato R Ueda S: Characteristics of fermentation yeast isolated from traditional Ethiopian honey wine, *ogol*. 2005;4(2):160–3. Reference Source

[17] Ivorra C Pérez-Ortín JE del Olmo M: An inverse correlation between stress resistance and stuck fermentations in wine yeasts. A molecular study. 1999;64(6):698–708. 10.1002/(SICI)1097-0290(19990920)64:6<698::AID-BIT9>3.0.CO;2-Z 10417219

[18] Rahman SS Hossain MM Choudhury N: Effect of Various Parameters on the Growth and Ethanol Production by Yeasts Isolated from Natural Sources. 2013;30(1–2):49–54. 10.3329/bjm.v30i1-2.28453

[19] Nasir A Rahman SS Hossain MM: Isolation of *Saccharomyces Cerevisiae* from Pineapple and Orange and Study of Metal’s Effectiveness on Ethanol Production. 2017;7(1):76–91. 10.1556/1886.2016.00035 28386473 PMC5372483

[20] Rahman SS Sarkar MK Islam MR: Isolation of yeasts from raisins and palm-juice and ethanol production in molasses medium. 2016;9(12). 10.17485/ijst/2016/v9i12/85509

[21] Rahman SS Hossain MM Choudhury N: Bioethanol fermentation from kitchen waste using *Saccharomyces cerevisiae* [version 1; referees: awaiting peer review]. 2018;7:512 10.12688/f1000research.14594.1

[22] Nofemele Z Shukla P Trussler A: Improvement of ethanol production from sugarcane molasses through enhanced nutrient supplementation using *Saccharomyces cerevisiae*. 2012;3(2):29–35. Reference Source

[23] Fakruddin M Quayum MA Ahmed MM: Analysis of Key Factors Affecting Ethanol Production by *Saccharomyces cerevisiae* IFST-072011. 2012;11(4):248–52. 10.3923/biotech.2012.248.252

[24] Khan AR Malek MA Choudhury N: Alcohol production from molasses and liquid sugar using some indigenous yeast isolates. 1989;6(1):37–42.

[25] Singh A Kuila A Adak S: Utilization of Vegetable Wastes for Bioenergy Generation. 2012;1(3):213–22. 10.1007/s40003-012-0030-x

[26] Lee CR Sung BH Lim KM: Co-fermentation using Recombinant *Saccharomyces cerevisiae* Yeast Strains Hyper-secreting Different Cellulases for the Production of Cellulosic Bioethanol. 2017;7(1): 4428. 10.1038/s41598-017-04815-1 28667330 PMC5493647

[27] Li YJ Lu YY Zhang ZJ: Co-fermentation of Cellulose and Sucrose/Xylose by Engineered Yeasts for Bioethanol Production. 2017;31(4):4061–7. 10.1021/acs.energyfuels.7b00032

[28] Casa-Villegas M Marín-Navarro J Polaina J: Synergies in coupled hydrolysis and fermentation of cellulose using a *Trichoderma reesei* enzyme preparation and a recombinant *Saccharomyces cerevisiae* strain. 2017;33(7):140. 10.1007/s11274-017-2308-4 28589508

[29] Promon SK Kamal W Rahman SS: Dataset 1 in: Ethanol production using vegetable peels medium and the effective role of cellulolytic bacterial (Bacillus subtilis) pre-treatment. 2018 Data Source 10.12688/f1000research.13952.1 PMC5968363 29899975

[30] Promon SK Kamal W Rahman SS: Dataset 2 in: Ethanol production using vegetable peels medium and the effective role of cellulolytic bacterial (Bacillus subtilis) pre-treatment. 2018 Data Source 10.12688/f1000research.13952.1 PMC5968363 29899975

