# Supplementary material for: Bioethanol production using vegetable peels medium and the effective role of cellulolytic bacterial (Bacillus subtilis) pre-treatment
Source: F1000Res. 2018 May 3;7:271. Originally published 2018 Mar 5. [Version 2] doi: 10.12688/f1000research.13952.2 (PMC5968363; doi:10.12688/f1000research.13952.2)
Supplement: Alcohol production from vegetable peels by yeast isolates SC1 and DJ1 at pH 6. Media pre-treated with Bacillus subtilis [file f1000research-7-16203-s0000.tgz › 40663f67-590f-4a6c-b678-b30476219ece_Dataset_1.docx]

**Dataset 1. Alcohol production from vegetable peels by yeast isolates SC1 and DJ1 at pH 6. Media pre-treated with *Bacillus subtilis***

| **Isolate** | **Defined Cellulosic Medium** | **Percentage of ethanol after 24 hours** | | | **Avg. Gm/L (w/v)** | **Percentage of ethanol after 48 hours** | | | **Avg. Gm/L (w/v)** |
| --- | --- | --- | --- | --- | --- | --- | --- | --- | --- |
|  |  | **First round** | **Second round** | **Avg.** |  | **First round** | **Second round** | **Avg.** |  |
| SC1 | Potato (150gm/1000ml) | 8.19 | 8.35 | 8.27 | 82.7 | 7.67 | 6.97 | 7.11 | 71.1 |
| DJ1 |  | 7.19 | 6.71 | 6.95 | 69.5 | 6.56 | 6.2 | 6.38 | 63.8 |
| SC1 | Papaya (150gm/1000ml) | 11.5 | 11.8 | 11.65 | 116.5 | 12.07 | 12.03 | 12.05 | 120.5 |
| DJ1 |  | 10.22 | 9.82 | 10.02 | 100.2 | 10.49 | 9.87 | 10.18 | 101.8 |
| SC1 | Cucumber (150gm/1000ml) | 14.25 | 12.97 | 13.61 | 136.1 | 13 | 13.46 | 13.23 | 132.3 |
| DJ1 |  | 9.51 | 9.33 | 9.42 | 94.2 | 9.68 | 8.24 | 8.96 | 89.6 |
| SC1 | Potato + Papaya (75gm + 75gm/1000ml) | 14.74 | 13.02 | 13.88 | 138.8 | 14.21 | 14.13 | 14.17 | 141.7 |
| DJ1 |  | 13.92 | 13.46 | 13.69 | 136.9 | 12.35 | 12.13 | 12.24 | 122.4 |
| SC1 | Potato + Cucumber (75gm + 75gm/1000ml) | 7.11 | 7.27 | 7.19 | 71.9 | 6.96 | 6.88 | 6.92 | 69.2 |
| DJ1 |  | 7.14 | 6 | 6.57 | 65.7 | 6.71 | 6.41 | 6.56 | 65.6 |
| SC1 | Cucumber + Papaya (75gm + 75gm/1000ml) | 8.51 | 8.07 | 8.29 | 82.9 | 9.35 | 8.89 | 9.12 | 91.2 |
| DJ1 |  | 7.32 | 6.92 | 7.12 | 71.2 | 7.28 | 7.22 | 7.25 | 72.5 |
