# Supplementary material for: Bioethanol production using vegetable peels medium and the effective role of cellulolytic bacterial (Bacillus subtilis) pre-treatment
Source: F1000Res. 2018 May 3;7:271. Originally published 2018 Mar 5. [Version 2] doi: 10.12688/f1000research.13952.2 (PMC5968363; doi:10.12688/f1000research.13952.2)
Supplement: Alcohol production from vegetable peels by yeast isolates SC1 and DJ1 at pH 6. Media without Bacillus subtilis pre-treatment [file f1000research-7-16203-s0001.tgz › 4dd52f09-347f-46cd-ba28-d47b11a2d83e_Dataset_2.docx]

**Dataset 2. Alcohol production from vegetable peels by yeast isolates SC1 and DJ1 at pH 6. Media without *Bacillus subtilis* pre-treatment**

| **Isolate** | **Defined Cellulosic Medium** | **Percentage of ethanol after 24 hours** | | | **Avg. Gm/L (w/v)** | **Percentage of ethanol after 48 hours** | | | **Avg. Gm/L (w/v)** |
| --- | --- | --- | --- | --- | --- | --- | --- | --- | --- |
|  |  | **First round** | **Second round** | **Avg.** |  | **First round** | **Second round** | **Avg.** |  |
| SC1 | Potato (150gm/1000ml) | 5.09 | 4.87 | 4.98 | 49.8 | 5.13 | 5.11 | 5.12 | 51.2 |
| DJ1 |  | 4.78 | 4.54 | 4.66 | 46.6 | 4.79 | 4.57 | 4.68 | 46.8 |
| SC1 | Papaya (150gm/1000ml) | 6.37 | 5.93 | 6.15 | 61.5 | 6.25 | 6.13 | 6.19 | 61.9 |
| DJ1 |  | 5.23 | 5.07 | 5.15 | 51.5 | 5.2 | 5.16 | 5.18 | 51.8 |
| SC1 | Cucumber (150gm/1000ml) | 5.77 | 5.63 | 5.7 | 57.01 | 5.75 | 5.51 | 5.63 | 56.3 |
| DJ1 |  | 5.43 | 5.49 | 5.46 | 54.6 | 5.26 | 5.24 | 5.25 | 52.5 |
| SC1 | Potato + Papaya (75gm + 75gm/1000ml) | 6.25 | 6.11 | 6.18 | 61.8 | 6.52 | 5.9 | 6.21 | 62.1 |
| DJ1 |  | 3.83 | 4.11 | 3.97 | 39.7 | 4.26 | 4.4 | 4.33 | 43.3 |
| SC1 | Potato + Cucumber (75gm + 75gm/1000ml) | 2.42 | 2.32 | 2.37 | 23.7 | 2.56 | 2.22 | 2.39 | 23.9 |
| DJ1 |  | 2.23 | 2.11 | 2.17 | 21.7 | 2.21 | 2.09 | 2.15 | 21.5 |
| SC1 | Cucumber + Papaya (75gm + 75gm/1000ml) | 4.54 | 4.36 | 4.45 | 44.5 | 4.69 | 4.45 | 4.57 | 45.7 |
| DJ1 |  | 3.02 | 2.84 | 2.93 | 29.3 | 3.03 | 2.99 | 3.01 | 30.1 |
