# Supplementary material for: Bioethanol production using vegetable peels medium and the effective role of cellulolytic bacterial (Bacillus subtilis) pre-treatment
Source: F1000Res. 2018 May 3;7:271. Originally published 2018 Mar 5. [Version 2] doi: 10.12688/f1000research.13952.2 (PMC5968363; doi:10.12688/f1000research.13952.2)
Supplement: Peer review history

# Peer review: Referee response for version 2

**DOI:** 10.5256/f1000research.16203.r33728

**Published:** 2018-05-24

**Authors:** Md Fakruddin

**Affiliations:** 1 Department of Molecular Physiology, Faculty of Life Sciences, Kumamoto University, Kumamoto, Japan

The manuscript can be approved in its present shape.

I have read this submission. I believe that I have an appropriate level of expertise to confirm that it is of an acceptable scientific standard.

# Peer review: Referee response for version 2

**DOI:** 10.5256/f1000research.16203.r33727

**Published:** 2018-05-17

**Authors:** Nazia Hossain

**Affiliations:** 1 Faculty of Integrated Technologies (FIT), University of Brunei Darussalam, Gadong, Brunei

I have read this submission. I believe that I have an appropriate level of expertise to confirm that it is of an acceptable scientific standard.

# Peer review: Referee response for version 1

**DOI:** 10.5256/f1000research.15166.r32524

**Published:** 2018-04-20

**Authors:** Md Fakruddin

**Affiliations:** 1 Department of Molecular Physiology, Faculty of Life Sciences, Kumamoto University, Kumamoto, Japan

1. Reference should be added in method sections.
2. Ethanol production was represented in both % and gm/L. Please use only one unit uniformly.
3. Long time fermentation (96 hr) should be done to ensure the efficiency of the isolate.
4. Introduction should be improved to address the objective of the study.
5. Compare your results with related published papers.
6. Please indicate the advantage of kitchen waste over more economical source such as molasses.
7. It is not clear what changes occur due to bacillus pretreatment.
8. Without large scale fermentation, the conclusion may be misleading.

I have read this submission. I believe that I have an appropriate level of expertise to confirm that it is of an acceptable scientific standard, however I have significant reservations, as outlined above.

# Response

**Published:** 2018-05-02

**Authors:** Shafkat Shamim Rahman

**Affiliations:** United Surgical (BD) Ltd., Bangladesh

We thank Dr. Fakruddin for his critical evaluation to improve the article. This report helps us to add few points to increase the quality of the article.

- We added new references in the methods section.
- In the preliminary submission, the results presented in percentage. In the revised article, gm/L (specific gravity reading) was also added to meet the editorial review requirement.
- Our previous studies (Rahman et al. 2013; Nasir et al. 2017) in the same lab showed the alcohol production reached the peak at 48 hours. After 96 hours, the rate either remain same or dwindled (possibly ethanol inhibition). So this study was designed for 48 hours.
- We added another objective in the introduction.
- We compared the results of tolerance tests with Teramoto et al. 2005, Ivorra et al. 1999 and results of fermentation to Nofemele et al. 2012, Khan et al. 1989, Rahman et al. 2013 and Nasir et al. 2017 in the discussion section. Now, we also added a comparison with related published paper.
- The advantage of kitchen waste over molasses is added at the end of the discussion.
- Figure 3 described the after effect of pretreatment, which resulted in increased ethanol production.
- Conclusions are ameliorated. If there is any specific recommendation, it would be helpful to conduct the revision.

# Peer review: Referee response for version 1

**DOI:** 10.5256/f1000research.15166.r31498

**Published:** 2018-03-16

**Authors:** Nazia Hossain

**Affiliations:** 1 Faculty of Integrated Technologies (FIT), University of Brunei Darussalam, Gadong, Brunei

1. Abstract
  - Kitchen waste might be a possible raw material for bioethanol production for clean technology according to the research result but it cannot be concluded as excellent source. Authors need to upgrade this view for the abstract (conclusion section).
2. Introduction
  - Please provide a brief description regarding the necessity of biofuel and bioethanol application in the beginning.
  - You may describe the common reactions what may take place for ethanol production from biomass and the role of microbes.
  - I suggest splitting first paragraph into 2 paragraphs and describing specific issues for each instead of mixing up.
3. Methods
  - Ok.
4. Result and Discussion
  - Did you isolate yeast from local resources due to economic purpose? If so, please mention it.
  - Sometimes fermentation might be effective after 48 hours (e.g. 72hr or 96hr). Please explain why this study have not tried upto 96 hr or above. Do your have any recommendation to do so in future?
  - Usually supplement addition (e.g. MgSO _4_) enhances fermentation process and increases overall bioethanol production. May you please elaborate why this study has not applied any nutritional component?
  - In discussion section, authors cited ‘Teramoto et al. (2005) demonstrated that members of Saccharomyces spp. can tolerate ethanol concentrations of up to 16.5%’ which is from 2005. Many studies have been performed with *Saccharomyces spp.* after 2005 what proved more than 50% bioethanol production. For instance, I suggest authors to check (Sugar and Bioethanol Production from Oil Palm
  Trunk (OPT) by Nazia Hossain & Rafidah Jalil).
  - Please provide few examples of comparative studies and compare your results with others since vegetable and fruits peels are very common sources of bioethanol generation worldwide.
5. Conclusion
  - The study result showed little amount of bioethanol production rate what is not favourable for economical view but environmental. So authors simply cannot assume that this procedure may be used for large-scale application without any specific modification or upgrade. In that case, author might recommend some factors to accelerate the production rate before scale-up.
  - I answered ‘Partly’ to the question ‘Are the conclusions drawn adequately supported by the results?’ because limitations are not mentioned in the conclusions.
6. Overall
  - Bioethanol is more appropriate than ethanol (In Title).
  - Grammar needs to be re-checked.
  - Many poorly structured sentences were being visible in the whole manuscript. Therefore, overall english should be improved.
  - I answered ‘Partly’ to the question ‘Is the work clearly and accurately presented and does it cite the current literature?’ as authors cited lot of old references while many upgraded experiments have been performed later on. I suggest authors to use references after 2000 only (especially for discussion part).

I have read this submission. I believe that I have an appropriate level of expertise to confirm that it is of an acceptable scientific standard, however I have significant reservations, as outlined above.

# Response

**Published:** 2018-05-02

**Authors:** Shafkat Shamim Rahman

**Affiliations:** United Surgical (BD) Ltd., Bangladesh

Thanks to Dr. Hossain for her constructive comments. We conducted a number of changes in response to her suggestions.

- Abstract (conclusion section) is corrected.
- The first paragraph of the introduction is modified by splitting and reorganizing.
- The reason to isolate yeast from local resources is added in the discussion.
- Our previous studies (Rahman et al. 2013; Nasir et al. 2017) in the same lab showed the alcohol production reached the peak at 48 hours. After 96 hours, the rate was either same or dwindled (possibly ethanol inhibition). So this study was designed for 48 hours.
- Addition of supplements usually increases the fermentation rate (Nasir et al. 2017), which was overlooked in the study design. We recommend adding this in future works.
- We express gratitude to enlighten us on the higher ethanol tolerance, which we believe will be helpful in future optimization studies.
- After 2000 references and comparison studies are added in the discussion.
- Limitations and recommendations are also added in the conclusion.
- The title of the article has also amended.
